# Quantitative Evaluation of Cardiac Cell Interactions and Responses to Cyclic Strain

**DOI:** 10.3390/cells10113199

**Published:** 2021-11-17

**Authors:** Richard Duc Hien Tran, Tessa Altair Morris, Daniela Gonzalez, Ali Hatem Salaheldin Hassan Ahmed Hetta, Anna Grosberg

**Affiliations:** 1 Edwards Lifesciences Center for Advanced Cardiovascular Technology, University of California, Irvine, CA 92617-2700, USA; rdtran1@uci.edu (R.D.H.T.); tessam@uci.edu (T.A.M.); gonzad14@uci.edu (D.G.); asalahel@uci.edu (A.H.S.H.A.H.); 2 Department of Biomedical Engineering, University of California, Irvine, CA 92617, USA; 3 Center for Complex Biological Systems, University of California, Irvine, CA 92697, USA; 4 NSF-Simons Center for Multiscale Cell Fate Research, University of California, Irvine, CA 92697, USA; 5 Department of Chemical and Biomolecular Engineering, University of California, Irvine, CA 92617, USA

**Keywords:** heart tissue organization, cell type classification, cyclic strain, intercellular junctions

## Abstract

The heart has a dynamic mechanical environment contributed by its unique cellular composition and the resultant complex tissue structure. In pathological heart tissue, both the mechanics and cell composition can change and influence each other. As a result, the interplay between the cell phenotype and mechanical stimulation needs to be considered to understand the biophysical cell interactions and organization in healthy and diseased myocardium. In this work, we hypothesized that the overall tissue organization is controlled by varying densities of cardiomyocytes and fibroblasts in the heart. In order to test this hypothesis, we utilized a combination of mechanical strain, co-cultures of different cell types, and inhibitory drugs that block intercellular junction formation. To accomplish this, an image analysis pipeline was developed to automatically measure cell type-specific organization relative to the stretch direction. The results indicated that cardiac cell type-specific densities influence the overall organization of heart tissue such that it is possible to model healthy and fibrotic heart tissue in vitro. This study provides insight into how to mimic the dynamic mechanical environment of the heart in engineered tissue as well as providing valuable information about the process of cardiac remodeling and repair in diseased hearts.

## 1. Introduction

The two dominant cell types in the myocardium are cardiomyocytes and cardiac fibroblasts; cardiomyocytes generate contractile force [1,2,3], while fibroblasts play vital roles in maintaining functions within the heart, such as extracellular matrix production, cardiac remodeling, cell–cell signaling, promoting blood vessel formation, and secretion of growth factors and cytokines [4,5,6]. In a healthy heart, cardiomyocytes and fibroblasts are organized along the direction of contraction [7,8,9]. However, in the event of myocardial infarction or other cardiac diseases, there is increased migration of fibroblasts into the regions of damaged tissue as well as changes to the morphology and viability of the myocytes [10,11,12,13,14,15]. The alterations in cellular composition and structure result in disorganization and loss of efficient heart function [16,17,18].

Although cellular disorganization, as well as a shift in the dominant cell type as a result of injury or remodeling, such as fibrosis, has been observed, the mechanisms that drive the organization of cardiomyocytes and fibroblasts and how they influence each other are not fully understood. Investigating the mechanisms responsible for organization in the heart is imperative to create accurate in vitro models of infarcted or diseased hearts, propose new pathways for treatment, and improve tissue engineering approaches such as cardiac patches [19,20,21]. To elucidate these mechanisms, cardiac cellular organization has been examined using a plethora of in vitro approaches [22,23]. In particular, one factor that is commonly implicated as a driving force for cellular organization is the application of strain to the cells [24,25,26,27,28]. Although several studies have shown that cells respond and organize when exposed to static/cyclic strains/stresses, the response is not consistent for all cardiac cell types [24,27,28,29,30,31]. In contrast to what is observed in healthy myocardium, where both cell types are present and co-oriented parallel to contraction, in isolated in vitro cultures that are exposed to uniaxial cyclic strain, fibroblasts orient approximately perpendicular to the strain direction [24,25,27], while cardiomyocytes orient approximately along the direction of mechanical stimulation [32]. A full understanding of the factors that may contribute to how cells organize in healthy heart tissue is essential for a slew of applications.

An unexplored factor that may be responsible for how cells organize in the heart is the interaction between the different cell types. In highly organized cardiac tissue, cardiomyocytes and fibroblasts are in direct contact with each other. To communicate, the cardiac cells are electrically and mechanically coupled via gap and adherens junctions, both heterogeneously (i.e., cardiomyocyte–fibroblast) and homogeneously (i.e., cardiomyocyte–cardiomyocyte and fibroblast–fibroblast) [33]. The most abundant gap and adherens junctions in the heart are connexin 40 (Cx40), connexin 43 (Cx43), and connexin 45 (Cx45), and N-cadherin (N-cad) [1,6,26,33,34,35,36,37]. Interestingly, by inhibiting or blocking a subset of these junctions, studies have shown inhibited cell–cell contact, adhesion, and signaling between cells [1,26,33,36,38,39,40,41]. Therefore, observing interactions between distinct cardiac cell types in confluent co-cultures through intercellular junctions or physical contact may be necessary to understand the guiding mechanisms for cardiac tissue reorganization in its native dynamic mechanical environment.

Image-based assays could be a powerful experimental tool to study interactions or behavioral differences between distinct cardiac cell types [42,43,44,45,46,47,48,49,50,51]. However, these assays often require the development of new image processing pipelines to extract information from the images that is relevant to the research question [45]. Indeed, studying confluent tissues with multiple cell types requires analysis that accurately and reliably distinguish between cell types in the same image. Thus, it is necessary to generate semantic segmentations of images, where each pixel in an image is associated with a classification corresponding to the cell type [48]. Many existing cell classification pipelines separate regions in an image based on differences in intensity and commonly make use of machine learning classifiers [43,47,48,49,50,51]; however, distinguishing mature striated myocytes from other cell types presents a unique challenge. Indeed, identifying mature striated myocytes requires not only recognizing the presence of a striated myocyte-specific stain but also examining the structures (i.e., sarcomeres) visualized by that stain. Additionally, there can be large variability in the development and thus appearance of the sarcomeres, which further complicates striated myocyte identification [52,53]. Therefore, it may be necessary to extract features that describe the spatial or statistical distribution of intensity in an image region, commonly referred to as image texture, which can be used by a machine learning classifier [54]. Thus, to accurately and reliably distinguish between distinct cardiac cell types in a single image and to study interactions between the cells in confluent co-cultures or sectioned myocardium, the use of advanced image processing tools will be required.

In this work, we created a system to study if different cardiac cellular compositions and intercellular junctions have an effect on heart tissue organization. To study this, we identified specific densities that are relevant to physiological states to examine how heart tissue organizes in response to cyclic strain. In addition, we investigated certain intercellular junctions that are abundant in the heart to elucidate their role in tissue organization. To quantify the differences in cellular organization under each experimental condition, we developed a classifier that is able to distinguish between cardiac cell types and quantified the orientational organization of the cell types separately. These experiments allowed us to elucidate the interaction between multiple cardiac cell types and several intercellular junctions to explain the organization behavior in healthy and diseased heart tissue.

## 2. Results

**Figure 1:**
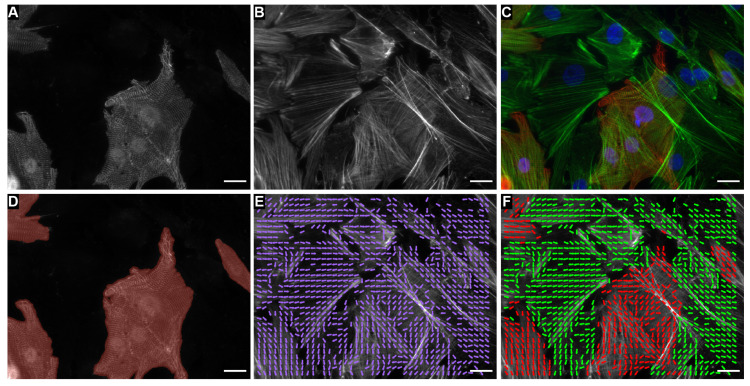
Co-culture of cardiomyocytes and fibroblasts labeled with (**A**) α-actinin, (**B**) actin, and (**C**) α-actinin (red), actin (green), and DAPI (blue). (**D**) Cells classified as mature cardiomyocytes are shaded red in the α-actinin labeled image shown in A. (**E**) The orientation of actin at each pixel is plotted as a purple arrow on top of the actin labeled image shown in B. (**F**) The orientation of actin at each pixel as shown in B but colored red for cardiomyocytes and green for fibroblasts. Scale bars: 20 µm.

**Figure A1:**
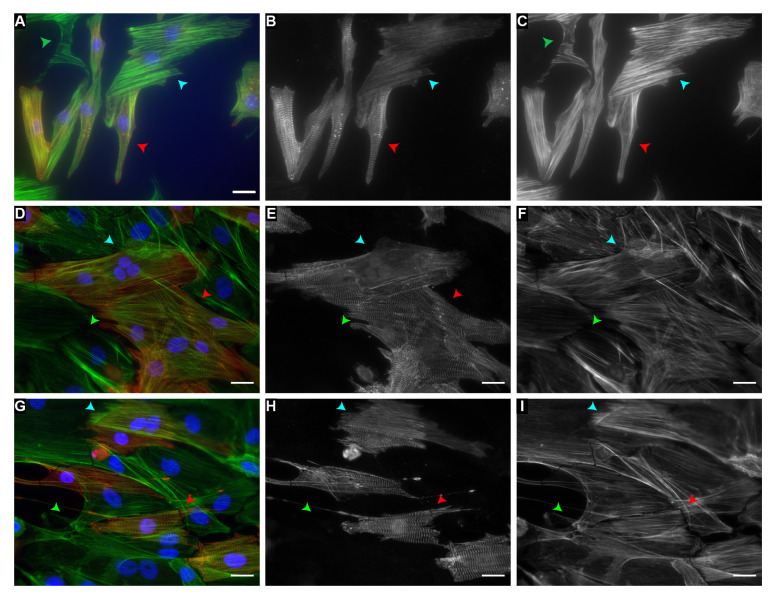
Co-culture of cardiomyocytes labeled with (**A**,**D**,**G**) α-actinin in red, actin in green, and DAPI in blue, (**B**,**E**,**H**) α-actinin, and (**C**,**F**,**I**) actin. In (**A**–**I**), the red arrowhead points to cardiomyocytes, the green arrowhead to fibroblasts, and the cyan to cells with α-actinin, but no identifiable sarcomere striations. Scale bars: 20 µm.

Studying how cardiomyocytes and fibroblasts organize in response to mechanical stimulation required the ability to reliably separate them in images and measure their individual orientation. While cellular orientation can be described by the orientation of actin fibrils in images (Figure 1B,E), it is difficult to establish which type of cell the actin belongs to without the use of additional stains. Therefore, the cardiac tissues were labeled with actin (Figure 1B,C), which appears in both cardiomyocytes and fibroblasts, as well as α-actinin, which only occurs in striated myocytes (Figure 1A,C). The identification was further complicated by qualitative observations of cells that were positive for the sarcomeric protein, α-actinin, but did not have any sarcomere striations (Appendix A Figure A1). Therefore, identifying mature or well-formed striated myocytes required not only recognizing the presence of a striated myocyte-specific stain (e.g., α-actinin) but also examining the structures (i.e., sarcomeres) visualized by that stain. As the phenotype of the cells with α-actinin, but no sarcomeric striations, cannot be identified as mature striated myocytes, we designated three semantic classes, “Fibroblast”, “Striated Myocyte”, and “Other” (Table 1). The classified α-actinin image (Figure 1D) was then used to distinguish actin belonging to fibroblasts from that belonging to cardiomyocytes. Once the actin orientation vectors (Figure 1E,F) were grouped according to their classification, the principal direction (i.e., director) and the spread in the distribution of the orientation vectors, also known as the orientational order parameter (OOP), were calculated (See the Materials and Methods Section 4.11 for more information). Thus, with the cell type-specific director and the OOP, we can quantify the predominant orientation of each cell type as well as the alignment between different cell types.

**Figure 2:**
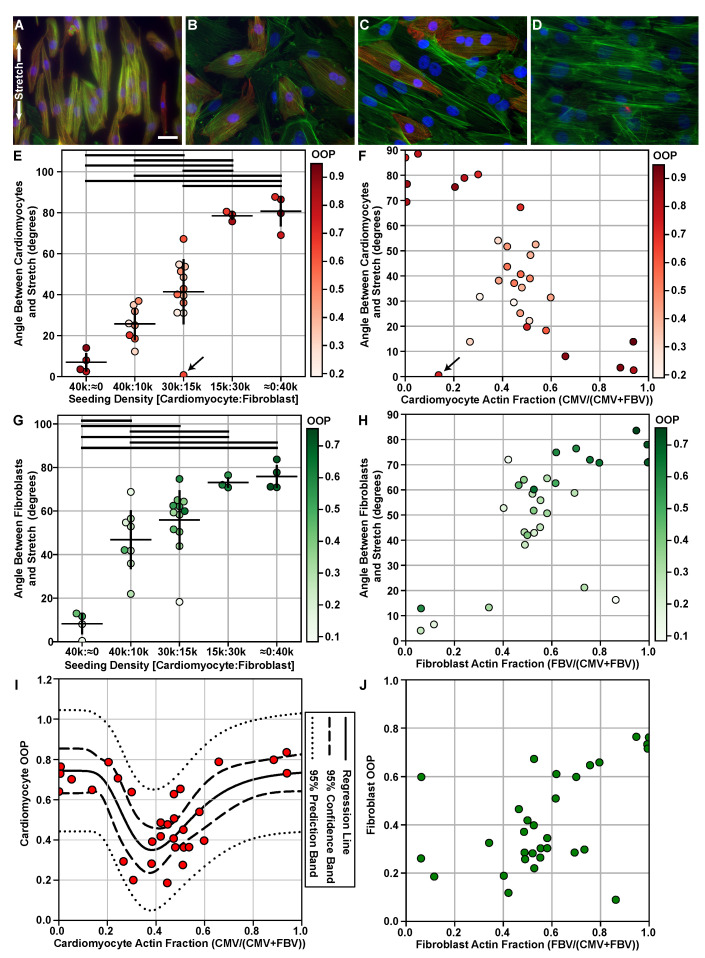
Cell type-specific actin orientation with respect to the direction of applied cyclic strain for different co-culture densities. Representative images of different experimental co-culture densities (**A**) 40k:≈0 (**B**) 30k:15k (**C**) 15k:30k (**D**) ≈0:40k labeled with α-actinin (red), actin (green), and DAPI (blue). (**E**) The average and standard deviation of the angle between the cardiomyocyte actin director and stretch director for each experimental seeding density. (**F**) The angle between the cardiomyocyte actin director and stretch director for the measured cell type actin fraction, i.e., the total number of cardiomyocyte actin vectors divided by the total number actin vectors. (**G**) The average and standard deviation of the angle between the fibroblast actin director and stretch director for each experimental seeding density. (**H**) The angle between the fibroblast actin director and stretch director for the measured cell type actin fraction, i.e., the total number of fibroblast actin vectors divided by the total number actin vectors. (**I**) Cardiomyocyte OOP as a function of actin fraction; fitted with a log-normal equation (p<0.05); regression line (solid), 95% confidence interval (dashed), and 95% prediction band (dotted). (**J**) Fibroblast OOP as a function of actin fraction. In (**A**,**B**), the arrows indicate a well with sparser than expected density. In (**E**–**J**), each point represents a single well, which is colored by the actin OOP for cardiomyocytes (**E**,**F**,**I**) or fibroblasts (**G**,**H**,**J**). Sample sizes of each experimental condition can be found in Table A1. In (**E**,**G**), horizontal bars indicate groups with significantly different means (p<0.05). All scale bars: 25 µm.

In order to understand the guiding mechanisms for cardiac tissue reorganization in a dynamic mechanical environment, cardiomyocytes and fibroblasts were co-cultured at different densities (Table 2 and Figure 2A–D) in a stretcher device and were exposed to 15% cyclic uniaxial strain at 1 Hz for 48 h. To prevent any bias, all of the data were preserved even though, in some cases, the seeding densities did not match the actual densities measured by the cell type actin fraction (Figure 2E–H). As a consequence, a few of the cultures were sparser than expected and resulted in the cells orienting to their preferential direction (i.e., Figure 2E,F, arrows). For confluent monocultures of cardiomyocytes, cells organized as previously observed in vitro [32], approximately parallel to the direction of stretch (Figure 2A,E at 40k:≈0). Similarly, fibroblasts seeded as a monoculture also organized as expected, approximately perpendicular to the direction of stretch (Figure 2D,G at ≈0:40k) [24,25,27]. However, for co-cultures, the principal direction of both cardiomyocyte and fibroblast actin relative to stretch changed depending on the dominant cell type (Figure 2E–H). At the 4:1, 2:1, and 1:2 cardiomyocyte to fibroblast seeding densities, cardiomyocytes gradually transitioned from the parallel direction to a direction perpendicular with respect to stretch (Figure 2E 40k:10k, 30k:15k, and 15k:30k, respectively). The OOP of the cardiomyocyte actin was also lower at the intermediate ratios, indicating that there was high variability in the alignment of the cardiomyocytes with respect to stretch (Figure 2I,J between 0.2 and 0.8). This is particularly evident at the 2:1 and 1:2 ratios where neighboring cardiomyocytes can be oriented in different directions when examined qualitatively (Figure 2E 30k:15k and 15k:30k, respectively). It was also observed that for cardiomyocytes and fibroblasts at the intermediate ratios, 4:1 and 2:1, where the influence exerted by the prevalent cell type was not dominant enough to dictate organization fully, the two cell types have different average direction with respect to stretch, (i.e., directors) (Figure 2E,G 40k:10k and 30k:15k, respectively). This suggests that how the cells are orienting is part of a stochastic process where which cells adhere to the culture first can influence the spread and amount of cell–cell contact. As seen in the intermediate densities with both cell types, the randomness can easily be distinguished from the highly organized edges (Figure 2F,H–J fractions <0.2 and >0.8).

The fibroblast orientation was also a function of the seeding densities. At seeding densities with higher amounts of fibroblasts, 1:2 and ≈0:1, the principal direction of cardiomyocytes generally followed the fibroblast-preferred orientation (close to perpendicular to stretch) and, thus, had a relatively high OOP (Figure 2G,H 15k:30k and ≈0:40k, respectively). The principal direction between fibroblasts and the stretch direction gradually decreased as there were relatively more cardiomyocytes (Figure 2G,H). However, unlike the cardiomyocytes, the fibroblast actin OOP decreased with the relative number of fibroblasts (Figure 2J). From these results, for both cardiomyocytes and fibroblasts, the presence of one another in co-cultures caused a deviation from their observed orientation in monocultures when exposed to cyclic strain.

(1) $$y=y_{0}+\frac{a}{x}\exp [-0.5{\left(\frac{\ln(\frac{x}{x_{0}})}{b}\right)}^{2}]$$

Cardiomyocyte organization as a function of cardiomyocyte actin fraction is well described by a log-normal equation ($R^{2}$ = 0.5554, Figure 2I). The data are well fitted as seen with the strong *p*-values (Table 3) and the 95% confidence and prediction bands. When cardiomyocytes occupied approximately 35% of the culture, this corresponded to an area with low organization indicated by the minimum of the regression line (Figure 2I). The tail ends of organization on the left and right of the fit represented areas with high organization where cardiomyocytes were guided by fibroblasts or by each other, respectively (Figure 2I < 0.2 and >0.8). In contrast, fibroblast OOP did not have an obvious minimum and could not be fitted similarly ($R^{2}$ = 0.3902, Figure 2J). From previous experiments with cardiomyocytes, it is known that fibroblasts are more dynamic and often spread into areas with no cells. As a result, fibroblasts can orient in the direction they move or spread even without guidance. Consequently, fibroblasts have more randomness to their organization, which is seen when examining fibroblast organization as a function of fibroblast actin fraction, especially at densities where there is low amounts of fibroblasts (Figure 2J fractions < 0.2). Thus, to understand organization of heart tissue, it is important to look at the OOP of cardiomyocytes and fibroblasts separately.

## 3. Discussion

In this work, we investigated cardiac tissue reorganization by examining co-cultures of the two dominant cell types in the myocardium, fibroblasts, and cardiomyocytes under the influence of cyclic strain to model both the cellular composition of the heart in health and pathology as well as the cardiac mechanical environment (Table 2) [1,2,3]. In order to measure the cell type-specific organization, we developed an image-processing pipeline to automatically classify images of tissues stained with α-actinin and to use the classification labels to separate the actin belonging to fibroblasts and striated myocytes (Figure 1). With the novel ability to automatically quantify the organization of actin for the individual cell types, we then evaluated the cell type-specific structure using organizational metrics, such as the OOP and the director (Figure 2E–J and Figure 4A–F).

The classifier developed for this work is capable of identifying mature sarcomeric striations in order to distinguish between different cardiac cell types and quantify the orientational organization of each cell type separately, which has never been done before. With the classifier, we were able to identify the organizational differences between cell types that would have been overlooked if only the organization of tissue as a whole was measured. While the analysis protocol we developed can be used to quantify cell type-specific morphology in tissues comprised of multiple cell types, there are several limitations and opportunities for improvement. One limitation is that it is not possible to accurately distinguish between overlapping cells (Figure 1). Appropriately rectifying overlapping cells would require a 3D imaging modality, such as acquiring serial optical sections. Furthermore, extension of the analysis protocol to images acquired using 3D imaging modalities would be required to interpret the results of future 3D culture experiments. However, our results are still relevant since the areas of overlap were rarely observed in these monolayer experiments (Figure 2A–D). Another limitation is that image regions were classified rather than segmenting individual cells and then classifying those. The advantage of this approach allowed for the quantification of cell type-specific actin organization rather than the orientation and organization of individual cells. While not directly relevant to what was measured for our study, this aspect of the analysis makes it not possible to distinguish between a cell with disorganized actin and multiple cells with well-organized actin that are oriented in different directions, which, for other studies, may be important to understand the mechanisms that caused cells that were exposed to the same mechanical and cellular composition to orient differently (Figure 2B,C). In the future, it may be interesting to consider how the maturity and formation of the cardiomyocytes influences their response rather than simply eliminating cells with α-actinin but no identifiable sarcomeric striations (Figure 1).

The heart is a complex organ that consists of many different cell types and structures, which makes it difficult to fully replicate in vitro [1,2,3,55,56,57,58,59]. Thus, separating and simplifying the components that contribute to heart function would facilitate the investigation of vital processes, such as cardiac remodelling and repair. In this spirit, our work utilized two of the most abundant cell types in the heart, fibroblasts and cardiomyocytes [1,2,3,55], to procure a general understanding of how organization in the heart is determined and transformed with and without pathology. Together, with the application of mechanical cyclic strain to these co-cultures, a simplified yet representative model of the myocardial environment was developed. In order to create a robust model, the experiments incorporated various densities to capture physiological relevant events in the heart (Table 2) [6,8,11,12,60].

With a range of different seeding densities, we were able to replicate organization that mimics healthy heart tissue and organization with low OOP that mimics what happens during fibrosis (Figure 2E–J). However, the densities at which the organization of healthy heart tissue occurs differ between our system and the in vivo environment. In the ventricles of the human heart, there is approximately twice the amount of cardiomyocytes when compared to fibroblasts [1,2,3,55]. However, the familiar organization found in healthy heart tissue did not appear until cardiomyocytes occupied approximately 80% of the culture in vitro (Figure 2I fractions > 0.8) [7,8,9]. One of the possible reasons for the difference observed in the cellular composition in vitro is that the heart has a laminar hierarchy, which consists of layers or “sheets” of muscle a few myocytes thick connected by collagen fibers [56,57,58]. As a result, the cells within the heart are surrounded by extracellular matrix proteins and others cells in all directions, increasing both extracellular matrix and cell–cell contact [56,57,58]. Even though monolayer cultures of cardiomyocytes and fibroblasts incorporated physiologically relevant densities and are representative of a single sheet within the laminar hierarchy (Table 2), the reduction in cell–cell or extracellular matrix contact could potentially explain the shift in the organizational order observed (Figure 2E–J). On the other hand, although the density at which organization of healthy heart tissue occurred was shifted in vitro, organization correlating to fibrotic tissue appeared at the expected density where fibroblasts occupied approximately 65% of the culture (Figure 2I,J). Based on our results, in order to model healthy or fibrotic heart tissue in vitro, approximate cell ratios of 5:1 and 1:2, respectively, should be utilized as these densities mimic the organization found in vivo (Figure 2I). Although our experiments incorporated a limited number of seeding densities, these densities translate into a range that covered the possible culture cell ratios (Figure 2F,H). However, in the future, more seeding ratios could be tested to drill down on any specific optimal cell culture ratios.

**Figure 3:**
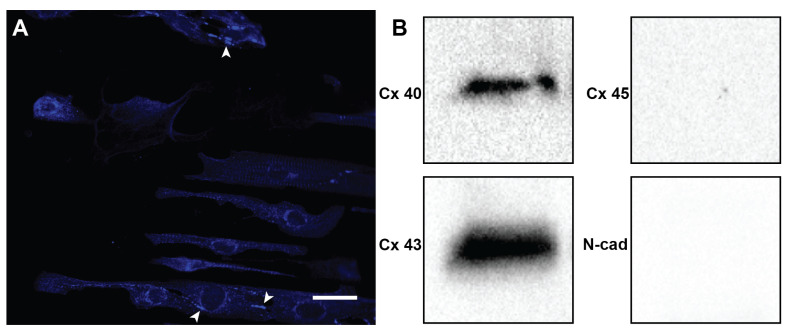
Intercellular junction presence. Cells stained for the presence of Connexin 43. (**A**) Example image a sampled stained for Connexin 43; Arrows indicate areas with noticeable connexin 43 junctions. Scale bar: 25 µm. (**B**) Western blot of connexins 40, 43, and 45 and N-cadherin.

**Figure 4:**
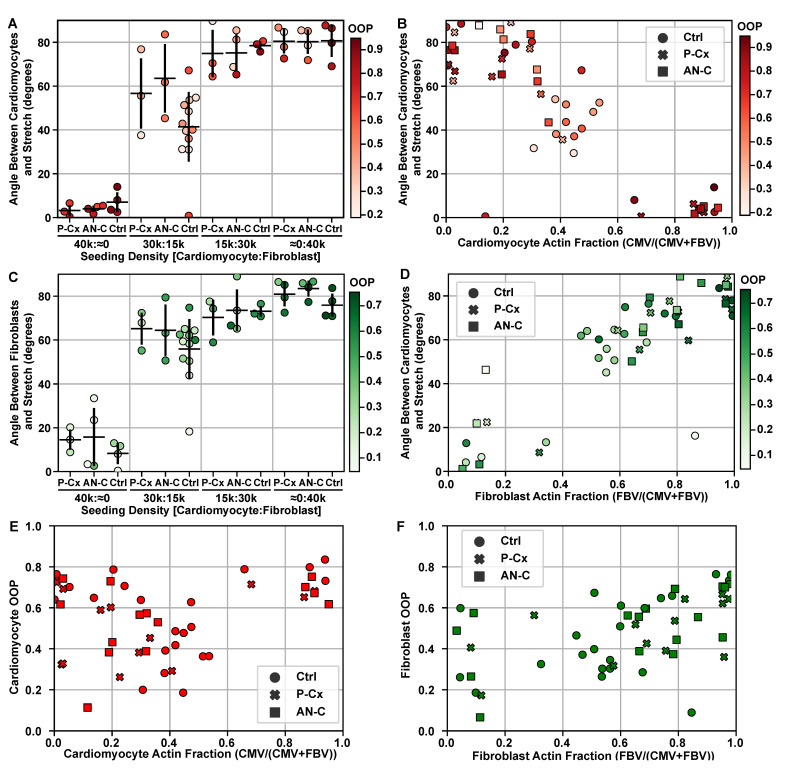
Co-cultures treated with drugs. Representative images of different experimental co-culture densities. ‘(**A**) The average and standard deviation of the angle between the cardiomyocyte actin director and stretch director for each experimental seeding density. (**B**) The angle between the cardiomyocyte actin director and stretch director for the measured cell type actin fraction, i.e., the total number of cardiomyocyte actin vectors divided by the total number actin vectors. (**C**) The average and standard deviation of the angle between the fibroblast actin director and stretch director for each experimental seeding density. (**D**) The angle between the fibroblast actin director and stretch director for the measured cell type actin fraction, i.e., the total number of fibroblast actin vectors divided by the total number actin vectors. (**E**) Cardiomyocyte OOP as a function of actin fraction. (**F**) Fibroblast OOP as a function of actin fraction. In (**A**–**F**), each point represents a single well, which is colored by the actin OOP for cardiomyocytes (**A**,**B**,**E**) or fibroblasts (**C**,**D**,**F**). Sample sizes of each experimental condition can be found in Table A1. In (**A**,**C**), horizontal bars indicate groups with significantly different means (p<0.05).

As cardiac cells are known to respond to mechanical strain [24,25,26,27], intercellular junctions that mechanically and electrically couple cells to one another are a potential factor that could play an influential role in controlling fibroblast and cardiomyocyte alignment in the heart. Within the heart, the predominant gap and adherens junctions are N-cad, Cx40, Cx43, and Cx45 depending on the area of the heart being examined [1,6,26,33,34,35,36,37]. Our experiments with cardiac cells originating from the ventricles of the heart confirmed the expression of connexins 40 and 43 with immunofluorescence imaging and Western blotting (Figure 3), which is consistent with previous studies [61,62]. Due to difficulty in quantifying the junctions through inflorescence, Western blots were used to judge the presence of the junctions instead. Interestingly, studies have shown that disrupting or blocking these junctions can inhibit cell–cell contact, adhesion, and signaling between cells [1,26,33,36,38,39]. However, in our experiments, using drugs to inhibit intercellular junctions of N-cad, Cx40, Cx43, and Cx45 resulted in no differences in the organization when compared to controls (Figure 4). Our results indicate that Cx40, Cx43, Cx45, and N-cad did not have a dominant effect on the reorganization of cardiomyocytes and fibroblasts in monolayer co-cultures exposed to cyclic strain. However, there are a few limitations that should be considered when interpreting these results, such as the effectiveness of Gap27 in comparison to knockdown studies and the differences in the abundance of intercellular junctions between monocultures and the laminar hierarchy of the heart [56,57,58,63,64]. In the future, staining on the single cell level for these junctions may be valuable to examine the exact effects of the cell–cell contact on cellular orientation; however, it is beyond the scope of these experiments. Additionally, though we saw no effect on organization of the cells by inhibiting the formation of these junctions by application of drugs in combination, future models should consider blocking these junctions separately to distinguish between any possible overlap of their influences.

Since there was no significant correlation between the intercellular junctions and organization of the two cardiac cell types, there may be other factors that can influence how these cells organize. While cells may communicate with paracrine factors, as evident from experiments with sparse and denser co-cultures, the cell–cell influence does not appear unless the two cell types are within proximity of one another (Figure 2B,C). Additionally, cardiomyocytes are active cells that constantly contract, but we have observed that the cardiomyocytes are organizing in their plastic state before they have mature, regular beating. What could affect cellular orientation in vivo are the physical constraints created by the highly organized and compact tissue structure. Furthermore, in the 3D laminar hierarchy, the cardiac extracellular matrix could aid in the guidance of the two cell types. Although our system simplifies the study of organization by limiting the factors that may influence organization in response to strain and cell–cell contact in 2D, cells may respond differently in 3D. Thus, extracellular matrix patterning, 3D assays, and more complex strain profiles representative of those found in vivo [65,66,67,68,69,70,71,72,73] would be the next candidates to incorporate into the model.

With the novel image processing pipeline created for this work, it is now possible to classify and quantify the organization of different cell types found in the heart. The cardiomyocytes and fibroblasts exhibited influences on the organization of one another when exposed to cyclic strain, especially in cases where the presence of one of the cell types was particularly dominant. However, in the intermediate densities, the influence exerted by the prevalent cell type was not dominant enough to dictate organization fully with each cell type trying to organize based on its preference. Furthermore, our data indicated that cell–cell contact via intercellular junctions was not a dominant mechanism that contributes to the organization of monolayered co-cultures of cardiomyocytes and fibroblasts. In this work, we were able to replicate the organization or lack thereof observed in healthy and fibrotic heart tissue, respectively, through the utilization of different cardiac cell types and mechanical strain. By establishing the cell ratios that would generate tissue resembling healthy or fibrotic cardiac tissue, we provide future models with working densities that could be used to further study cardiac repair and reorganization in vitro.

## 4. Materials and Methods

### 4.1. Cardiomyocyte and Fibroblast Harvest

All animals for the study were treated according to the Institutional Animal Care and Use Committee of University of California, Irvine guidelines (IACUC Protocol No. 2013-3093). The recommendations of the NIH Guide for the Care and Use of Laboratory Animals were followed, and the experiments were also in accordance with existing federal (9 CFR Parts 1, 2, and 3), state, and city laws and regulations governing the use of animals in research and teaching. Harvest was performed with a previously established protocol [53]. Briefly, 2-day-old neonatal Sprague–Dawly rat pups (Charles River Laboratories Wilmington, MA, USA) were euthanized by decapitation, and the ventricular myocardia were extracted. Cardiomyocytes were then isolated from the ventricular tissue as described previously [74,75,76,77]. Ventricular tissue were then washed with Hanks’ balanced salt solution buffer (HBSS; Life Technologies, Carlsbad, CA, USA) and then incubated overnight (12 h) at 4 ${}^{\circ }C$ in a 1 mg/mL trypsin solution (Sigma Aldrich, Inc., Saint Louis, MO, USA) dissolved in HBSS. After incubation, the trypsin solution was neutralized with warm M199 culture media (Invitrogen, Carlsbad, CA, USA) supplemented with 10% fetal bovine serum (FBS; ThermoFisher, Grand Island, NY, USA). The tissue was then washed four times with 1 mg/mL collagenase type II (Worthington Biochemical Corporation, Lakewood, NJ, USA) dissolved in HBSS. The isolated cell solutions were centrifuged at 1200 rpm for 10 min and re-suspended in chilled HBSS before being centrifuged again at 1200 rpm for 10 min. The cells were then re-suspended in warm 10% FBS M199 culture media and purified through three consecutive preplates. The final solution of cardiomyocytes were collected and then seeded at the required densities with or without fibroblasts (Table 2). Cardiac fibroblasts remaining within the preplates were kept and passaged once for experiments. The cardiac fibroblasts were cultured in the same 10% FBS M199 culture media. The preplates were then passaged at 80–100% confluency, using 0.05% trypsin (Fisher Scientific, Hanover Park, IL, USA). Once confluent, the cardiac fibroblasts were trypsinized, collected, counted, and seeded at the specified densities (Table 2).

### 4.2. Stretcher Experiments

MechanoCulture FX-2 (CellScale, CDN), a stretcher device, was used to apply cyclic stretch to the cells. The actuator was programmed to execute manufacturer specified 15% uniaxial cyclic stretch at 1 Hz for 48 h. Seeding the stretcher followed a previously established protocol [24]. Briefly, the wells were initially washed and primed with phosphate-buffered saline (PBS, ThermoFisher, Grand Island, NY, USA). A 0.05 mg/mL fibronectin solution was then added to each well (Fisher Scientific, Hanover Park, IL, USA) and incubated overnight at 4 °C. The wells were washed once more with PBS to remove excess fibronectin before adding a 300 µL solution of cells, culturing media, and drug depending on the experimental condition. The cells were placed inside an incubator with 5% CO2 at 37 °C for the duration of the experiment. After 30 min post seeding, the stretching protocol was initiated at the specified 15% uniaxial cyclic stretch at 1 Hz for 48 h. At the 24 h time point, the media were changed with culture media with or without drugs depending on the experimental condition. Once the whole 48 h cycle was completed, the cells were then fixed and immunostained.

### 4.3. Inhibiting Intercellular Junctions

To inhibit connexin 40, connexin 43, and connexin 45 intercellular formations, mimemtic peptides 40Gap27 (sequence SPRTEKNVFIV), 37/43Gap27 (sequence SRPTEKTIFII), and 45Gap27 (sequence SPRTEKTIFLL) were used, respectively. N-cadherin intercellular formation was inhibited with anti-N-cadherin mouse monoclonal antibody (Milipore Sigma, St. Louis, MO, USA). Each of the peptides and the antibody were used at the concentrations of 300 µM. The peptides and antibody where dissolved in 10% FBS M199 media. A solution with similar concentrations respective to each condition was used for the media change after the first 24 h of stretch.

### 4.4. Fixing and Immunofluorescent Staining

The cells were fixed with a solution of 4% paraformaldehyde (VWR, Radnow, PA, USA) and 0.05% Triton X-100 (Sigma-Aldrich, Saint Louis, MO, USA) for 15 min. Each well was washed three times with PBS for 5 min after fixing. The cultures were then stained for nuclei (4,6-diamidino-2-phenylindole dihydrochloride, DAPI, ThermoFisher, Grand Island, NY, USA), actin (Alexa Fluor 488 Phalloidin, ThermoFisher, Grand Island, NY, USA), and sarcomeric α-actinin (Mouse Monoclonal Anti α-actinin; Sigma Aldrich, Inc., St. Louis, MO, USA). The wells were then washed with PBS three times to remove excess stain. Secondary staining was completed with goat anti-rabbit IgG secondary antibodies (Alexa Fluor 633, ThermoFisher, Grand Island, NY, USA). The wells were once more washed 3 times with PBS to remove excess staining. Afterwards, the wells were punched out with a commercially purchased metal square hole puncher and mounted onto glass microscope slides. ProLong Gold Antifade Mountant (ThermoFisher, Grand Island, NY, USA) was applied, and a microglass coverslip was placed on top to cover and seal the sample. Connexin 43 staining followed a similar protocol with just the addition of primary Connexin 43 Antibody (Cell Signaling, Danvers, MA, USA) and goat anti-Mouse IgG secondary antibody (Cyanine5, ThermoFisher, Grand Island, NY, USA). All staining was completed at a dilution of 1:1000 in PBS.

### 4.5. Imaging and Data Acquisition

The samples were mounted at 90° and imaged with an IX-83 inverted motorized microscope (Olympus America, Center Valley, PA, USA). Images were taken using an UPLFLN 40x oil immersion objective (Olympus America, Center Valley, PA, USA) and a digital CCD camera ORCA-R2 C10600-10B (Hamamatsu Photonics, Shizuoka Prefecture, Japan). Ten fields of views randomly selected for each sample and imaged at 40x magnification (6.22 µm/pixel). Connexin 43 images were obtained with the Olympus Fluoview FV3000 microscope (Olympus America, Center Valley, PA, USA) also using a UPLSAPO 40x silicone oil immersion objective (Olympus America, Center Valley, PA, USA).

### 4.6. Western Blotting

The western blot was carried out with a previously established protocol [78,79]. Briefly, the cells were washed with PBS once before they were exposed to a solution of RIPA lysis buffer and 1% protease inhibitor (ThermoFisher, Grand Island, NY, USA). Twenty micrograms of the collected lysis solution was added to equal amounts of Laemmli buffer supplemented with 5% 2-mercaptoethanol and boiled at 95 °C for 10 min before loading into a well of 4–15% mini-PROTEANTM precast gels (Bio-Rad, Hercules, CA, USA). When gel electrophoresis was completed, the proteins were blotted onto nitrocellulose membranes using the iBlot dry blotting system (ThermoFisher, Grand Island, NY, USA). After electroblotting, the blots were blocked overnight at 4 °C using 5% nonfat milk. The blot was then stained for 1 h at RT with connexin 40 polyclonal antibody (ThermoFisher), connexin 43 antibody (Cell Signaling), 45 polyclonal antibody (ThermoFisher), and monoclonal anti-N-cadherin antibody (Sigma Aldrich) as primaries. After the first incubation with primaries, TBST was used to wash the blots for 15 min before further incubation with horseradish peroxidase-conjugated secondary for 1 h at RT. The blots were washed once more with TBST for 15 min after secondaries. For imaging, the blots were incubated in SuperSignal West Femto Maximum Sensitivity Substrate (ThermoFisher, Grand Island, NY, USA) for 5 min before imaging the blot using Bio-Rad ChemiDoc XRS+ with Image Lab software.

### 4.7. Statistical Analysis

To determine statistical significance, one-way analysis of variance (ANOVA) with Tukey’s Test was performed in Python 3.7.7 using the module statsmodels. A *p*-value less than 0.05 was considered significant.

### 4.8. Log-Normal Fit

To fit an equation to the cardiomyocyte OOP as a function of cardiomyocyte actin fraction, Regression Wizard of SigmaPlot was used. Nonlinear regression with a log-normal, 4-parameter equation was utilized with no constraints, and the fit converged after 29 iterations. Normality test (Shapiro–Wilk) was significant with a *p*-value less than 0.0001.

### 4.9. Image Classification

In order to distinguish between the background and foreground, manually labeled α-actinin images were entropy filtered, standard deviation filtered, range filtered, and Gaussian filtered with σ=5. The filtered and labels were subsampled, such that each class (i.e., “Background” and “Foreground”) was equally represented in the training data, and approximately 2.5% of each image was included in training. A total of 14 alpha-actinin images were manually labeled as ”Background”, ”Striated Myocyte”, or ”Other” and 2.5% of the total number of pixels (1024 × 1344 × 0.025) were included in the training data. Although the data were unbalanced, an equal number of pixels for each class was used for training. The MATLAB function fitctree, with the hyperparameters automatically optimized, was used to create the classifier that had an accuracy of 95.87% for the training data. Once the image foreground had been segmented, the foreground regions were classified as either “Striated Myocyte” or “Other”. To achieve this, each α-actinin image was broken up into 200 “super pixels,” which are groups of pixels with similar values, determined using a simple linear iterative clustering algorithm [80]. The orientation of the foreground objects was then computed after smoothing the images with a Gaussian kernel with σ=5. Striations in the images were identified by top hat filtering the anisotropic diffusion filtered images with the same method previously published by others Morris et al. [53], Persson et al. [81], Weickert [82], Perona and Malik [83], Kroon and Slump [84]. The top hat filtered image was then binarized using adaptive thresholding. In order to isolate sarcomeric z-line striations, the orientation of the striations was compared with the object orientation, using an approach similar to that described in Morris et al. [53]. The orientation of the striations was compared with the principal orientation of the object at each super pixel by taking the dot product and identifying striations that had a dot product value less than 0.8. Super pixel regions that were at least 10% covered in perpendicular striations were considered candidate striated myocyte regions. Additionally, to remove super pixel regions that did not contain enough striations, the top hat filtered image was binarized using global thresholding, and super pixels that consisted of less than 5% positive pixels were not considered striated myocyte regions.

### 4.10. Nuclei Segmentation and Cell Type Classification

Nuclei in DAPI-stained images were segmented using the ImageJ/FIJI plugin for “StarDist” [85]. The segmented nuclei images were loaded into MATLAB and compared with the classification labels generated for the α-actinin images. The α-actinin classification label associated with each individual nucleus was determined based on most common (i.e., mode) classification label that occurred within the boundary of the individual nucleus. If the most common classification label did not account for at least a threshold proportion of the nuclear boundary, the nucleus was not considered associated with a classification label. In this analysis, the threshold proportion was set to 0.4.

### 4.11. Cell Type Orientation Analysis

The orientation of actin at each pixel was calculated in order to determine the overall organization of each cell type as described previously [22,86]. Briefly, images were filtered with a Gaussian kernel and then standardized to have zero mean and unit standard deviation [87]. The orientation was then estimated using a least mean square orientation estimation algorithm [87,88]. Once the orientation vectors were computed for each pixel, the results of the classifier were used to separate the actin belonging to cardiomyocytes and fibroblasts. As described previously [53,74,89], the orientational order parameter (OOP) and principal direction (director) [90,91] of the orientation vectors, $\vec{r}\left(x,y\right)$, were quantified from the structure tensor T,

(2) $${\hat{T}}_{i}= [\begin{array}{cc} r_{i,x}r_{i,x} & r_{i,x}r_{i,y}\\ r_{i,x}r_{i,y} & r_{i,y}r_{i,y} \end{array}] .$$

The OOP is the maximum eigenvalue of T, and the director is the eigenvector corresponding to the maximum eigenvalue of tensor T. The angle (θ) between two unit vectors, $\vec{p}$ and $\vec{q}$, (e.g., the stretch direction and the director of either the cardiomyocyte actin or the fibroblast actin) was computed by taking the inverse cosine of the dot product between the two unit vectors

(3) $$\theta =\cos^{-1}\left(\vec{p}\;\cdot \;\vec{q}\right).$$

## Figures and Tables

**Table 1:** Description of semantic classes.

Class	α-Actinin Description	Actin Description
Fibroblast	no α-actinin	actin fibrils
Striated Myocyte	α-actinin sarcomere striations	actin fibrils
Other	α-actinin, but no sarcomere striations	actin fibrils
Background	no α-actinin	no actin fibrils

**Table 2:** Co-culture cell type ratios.

CM:FB Seeding Ratio	Description	Relevance
≈0:1	Fibroblast dominant	Recapitulate published results
1:2	Fibroblast dominant	Intermediate; beginning of injury/inflammation
2:1	Cardiomyocyte dominant	Physiologically relevant
4:1	Cardiomyocyte dominant	Rare fibroblast
1:≈0	Cardiomyocyte dominant	Recapitulate published results

**Table 3:** Log-normal fit details.

Variable	Description or Coefficient	Significance
x	Cardiomyocyte actin fraction	N/A
y	Cardiomyocyte OOP	N/A
a	−0.1605	<0.0001
b	0.3504	0.0006
$x_{0}$	0.4334	<0.0001
$y_{0}$	0.7437	<0.0001
$R^{2}$		0.5554

**Figure A2:**
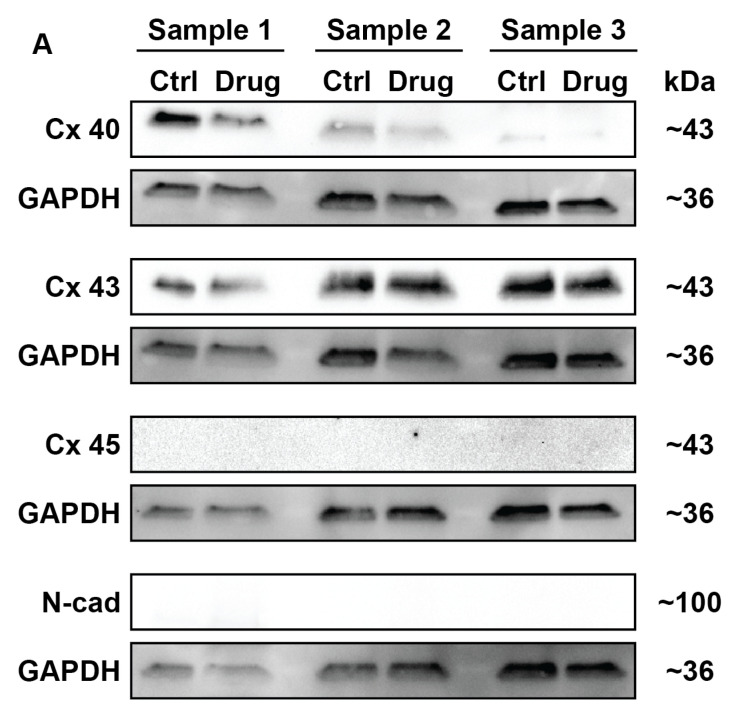
Western blot of three different experimental runs (Samples 1, 2, and 3) showing expression of each protein with (Drug) and without drugs (Ctrl). Each junction protein was run on a separate gel with three samples from different experimental runs on each gel.

## Data Availability

The data presented in this study are openly available on Dryad at https://doi.org/10.7280/D1PX16 (accessed on 26 August 2021).

## Appendix A

**Table A1:** Details of each seeding condition.

CM:FB Seeding Density	Drug Condition	Sample Size
40k:≈0	P-Cx	3
40k:≈0	AN-C	4
40k:≈0	Ctrl	4
40k:10k	Ctrl	8
30k:15k	P-Cx	3
30k:15k	AN-C	3
30k:15k	Ctrl	12
15k:30k	P-Cx	3
15k:30k	AN-C	4
15k:30k	Ctrl	3
≈0:40k	P-Cx	4
≈0:40k	AN-C	4
≈0:40k	Ctrl	4
